# Effect of colestimide on the concentrations of polychlorinated dibenzo-*p*-dioxins, polychlorinated dizenzofurans, and polychlorinated biphenyls in blood of Yusho patients

**DOI:** 10.1186/s12940-016-0150-z

**Published:** 2016-06-04

**Authors:** Takashi Todaka, Akinori Honda, Masami Imaji, Yoshiko Takao, Chikage Mitoma, Masutaka Furue

**Affiliations:** Kitakyushu Life Science Center, Public Interest Incorporated Foundation, Nakabarushinmachi 1-4, Tobata-ku, Kitakyushu-shi, Fukuoka 804-0003 Japan; Fukuoka Institute of Health and Environmental Sciences, 39, Mukaizano, Dazaifu-shi, Fukuoka 818-0135 Japan; Research and Clinical Center for Yusho and Dioxin, Kyushu University Hospital, Maidashi 3-1-1, Higashi-ku, Fukuoka 812-8582 Japan; Department of Dermatology, Graduate School of Medical Sciences, Kyushu University, Maidashi 3-1-1, Higashi-ku, Fukuoka 812-8582 Japan

**Keywords:** PCDDs, PCDFs, PCBs, Yusho, Colestimide, Blood concentration

## Abstract

**Background:**

Oral colestimide was reported to lower the concentration of PCDDs, PCDFs, and PCB in the blood of humans. A pilot study showed that the arithmetic mean total TEQ concentrations of PCDDs, PCDFs, and PCBs in the blood of subjects after the trial decreased approximately 20 % compared to pre-trial levels, suggesting that colestimide could decrease human dioxin levels. We designed the current clinical trial study based on this information. In this study, we examined whether colestimide could reduce the individual congener concentrations of PCDDs, PCDFs, and PCBs in the blood of Yusho patients.

**Methods:**

Out of the 36 Yusho patients who participated in the clinical trial, 26 patients self-administered colestimide 3 g/day orally for 6 months. The concentrations of PCDDs, PCDFs and PCBs in the blood of 26 Yusho patients before the trial were compared with those after the trial.

**Results:**

The arithmetic mean total TEQ concentrations of PCDDs, PCDFs, non-*ortho* PCBs, and mono-*ortho* PCBs in the blood of the 26 Yusho patients before and after the clinical trial were 42–303 (mean: 130, median: 120) and 43–283 (mean: 132, median: 118) pg TEQ/g lipid, respectively. The sums of the concentrations of 58 PCB congeners measured in the blood of Yusho patients before and after the trial were 321–2643 (mean: 957, median: 872) and 286–2007 (mean: 975, median: 806) ng/g lipid, respectively, indicating that the concentrations of PCDDs, PCDFs, and PCBs after the trial were almost the same as those before the trial. Among congeners of PCDDs, PCDFs, dioxin-like PCBs, and non-dioxin-like PCBs, most congeners of these compounds did not show a statistically significant decrease after the trial.

**Conclusion:**

Colestimide may not be beneficial in reducing the high blood levels of dioxin-like compounds in Yusho patients.

## Background

The 1968 Yusho poisoning accident affected over 1800 people in western Japan [1]. Since the Yusho outbreak, the National Study Group for the Therapy of Yusho has carried out medical care and health examinations of patients affected [2]. In 2001, the measurement of PCDDs, PCDFs, and non-*ortho* PCBs in the blood became possible using small amounts of blood collected from participants during annual medical examinations [3–5]. We have measured the concentrations of PCDDs, PCDFs, and dioxin-like PCBs in the blood collected from Yusho patients in medical health examinations since 2002 [6–8]. Moreover, we have conducted a congener-specific analysis of non-dioxin-like PCBs in the blood of these patients since 2004 [9, 10]. Based on these results, we previously reported that Yusho patients continue to have higher concentrations of PCDFs in their blood than unaffected people, and that concentration of PCDFs in the blood is significantly correlated with the intensity of Yusho symptoms [11, 12].

Development of effective therapy to reduce the concentrations of PCDDs, PCDFs, and PCBs in the blood of Yusho patients could improve the health care of these patients. With regard to promoting the excretion of lipophilic contaminants stored in the human body, several studies of dietary supplements such as cholestyramine, mineral oil, hexadecane, and dietary fiber have been reported using laboratory animals [13–16]. In addition, another study reported the enhancing effect of non-absorbable lipid substitute olestra on fecal excretion of PCDDs, PCDFs, and PCBs in the human body [17, 18]. Our study group previously conducted a clinical trial to reduce the concentrations of PCDDs, PCDFs, and PCB in the blood of Yusho patients using cholestyramine and rice bran fiber [19, 20]. However, beneficial clinical effects could not be confirmed due to the short trial period.

Colestimide, a 2-methylimidozarol-epichlorohydrin polymer, is widely used to lower serum cholesterol levels in Japan. Recently, oral colestimide was reported to lower the concentration of PCDDs, PCDFs, and PCB in the blood of humans [21, 22]. A pilot study showed that the arithmetic mean total TEQ concentrations of PCDDs, PCDFs, and PCBs in the blood of subjects after the trial decreased approximately 20 % compared to pre-trial levels, suggesting that colestimide could decrease human dioxin levels [21, 22]. We designed the current clinical trial study based on this information. In this study, we examined whether colestimide could reduce the individual congener concentrations of PCDDs, PCDFs, and PCBs in the blood of Yusho patients.

## Methods

### Sampling

The trial protocol was approved by the institutional ethics committee of Kyusyu University Hospital. Patients who fulfilled the diagnostic criteria for Yusho established by the National Study Group for the Therapy of Yusho were eligible for this study. Patients were recruited at explanatory meetings conducted in Fukuoka and Nagasaki Prefectures. 50 Yusho patients were enrolled in this clinical trial, and 14 patients refused to participate. The remaining 36 patients participated in the trial. Informed consent was obtained for study participation. The patients self-administrated colestimide 3 g/day orally for 6 months. Out of the 36 Yusho patients who participated in the clinical trial, 26 patients completed the trial. The 26 patients ranged in age from 60 to 87 years (mean: 72.9, median: 72.5). Among the 26 patients, there were 13 men (age range 60–87 years; mean: 73.1, median: 74.0) and 13 women (age range 61–81 years; mean: 72.8, median: 72.0). The blood samples examined in this study were collected between April 4, 2008 and July 15, 2009. After collection, the blood samples were stored at 4 °C until analyses.

### Materials

Native congeners of PCDDs, PCDFs, dioxin-like PCBs, and non-dioxin-like PCBs were purchased from Wellington Laboratories (Guelph, Canada). [^13^C_12_]–congeners of PCDDs, PCDFs, dioxin-like PCBs, and non-dioxin-like PCBs as internal standards, were also purchased from Wellington Laboratories. An active carbon column was prepared as follows: active carbon was purchased from Nacalai Tesque (Kyoto, Japan), refluxed 3 times with toluene for 1 h, and dried in vacuum, after which 500 mg of the active carbon was mixed with 500 g of anhydrous sodium sulfate (Wako Pure Chemical Industries, Ltd., Tokyo, Japan). A silver nitrate/silica gel was purchased from Wako Pure Chemical Industries, Ltd. All reagents and solvents used in this experiment were of the analytic grade of dioxin that is commercially available.

### Analysis of PCDDs, PCDFs, and PCBs

The extraction and purification of PCDDs, PCDFs, dioxin-like PCBs, and non-dioxin-like PCBs from blood samples were performed using a previously reported method [5, 9]. Concentrations of PCDDs, PCDFs, and dioxin-like PCBs and concentrations of 58 non-dioxin-like PCB congeners were determined by a previously reported method [5, 9].

### Quality control

To evaluate the accuracy and reliability of the analysis of PCDDs, PCDFs, dioxin-like PCBs, and non-dioxin-like PCBs, our laboratory prepared human blood samples and conducted quality control studies of the analysis of PCDDs, PCDFs, and dioxin-like PCBs in 2007, 2009, 2011, and 2013 and non-dioxin-like PCBs in 2008, 2010, 2012, and 2014. Each quality control study involved the participation of various laboratories that perform measurements for these compounds in human blood in Japan. In each quality control study, our results were compared with those of participating laboratories, and tests confirmed that the average variation among values obtained by each organization performing the analysis was all within 10 %. These results indicated that our laboratory’s analytical methods regarding PCDDs, PCDFs, dioxin-like PCBs, and non-dioxin-like PCBs in human blood provided accurate results.

### Data analysis

To estimate the TEQ concentrations, we introduced ND (less than the detection limit) values to half values of the detection limit and calculated based on the TEF values proposed by the WHO [23]. The statistical analysis was conducted using Wilcoxon signed-rank test in the software programs from Statistics Package for Social Sciences (version 22; IBM Armonk, NY, USA). Significant probabilities (*p* values) were calculated for the respective number of samples analyzed.

## Results

The objective of the present study was to evaluate the effectiveness of colestimide on the individual congener concentrations of PCDDs, PCDFs, and PCBs in blood of Yusho patients. Of the 36 Yusho patients who began the trial, 9 patients stopped administrating colestimide due to serious adverse effects, constipation or abdominal distension. Of the 27 remaining patients, we failed to collect a posttreatment blood sample from one patient due to cancellation of hospital visit. The individual congener concentrations of PCDDs, PCDFs and PCBs in the blood of 26 Yusho patients before the trial were compared with those after the trial (Tables 1 and 2).

**Table 1:** Effect of colestimide on the individual congener concentrations of PCDDs, PCDFs, and dioxin-like PCBs in the blood of Yusho patients

Congeners	Concentration (pg/g lipid)										*p* Values
	Before the clinical trial					After the clinical trial					
	Mean	Median	SD	Minimum	Maximum	Mean	Median	SD	Minimum	Maximum	
2,3,7,8-TetraCDD	1.8	1.7	0.9	0.5	4.0	2.0	1.8	1.2	0.5	4.7	0.083
1,2,3,7,8-PentaCDD	14	14	4.9	6.6	23	14	12	6.1	6.1	27	0.675
1,2,3,4,7,8-HexaCDD	3.1	3.0	1.7	1.0	7.1	3.3	3.2	1.7	1.0	6.9	0.053
1,2,3,6,7,8-HexaCDD	62	53	36	16	183	63	55	34	15	164	0.258
1,2,3,7,8,9-HexaCDD	5.5	4.5	5.2	2.1	29	5.7	3.9	6.0	1.0	31	0.770
1,2,3,4,6,7,8-HeptaCDD	55	47	25	21	113	52	43	27	20	143	0.137
OctaCDD	699	606	281	323	326	670	543	309	305	1610	0.118
Total PCDD	841	739	315	413	1525	811	688	346	382	1850	0.144
2,3,7,8-TetraCDF	2.8	2.7	1.3	0.5	5.5	2.7	2.6	1.4	0.5	5.8	0.427
1,2,3,7,8-PentaCDF	1.3	1.1	0.9	0.5	3.5	1.5	1.2	1.1	0.5	4.4	0.554
2,3,4,7,8-PentaCDF	241	191	158	48	636	242	205	158	49	613	0.732
1,2,3,4,7,8-HexaCDF	64	51	56	7.8	227	64	52	56	8.1	207	0.990
1,2,3,6,7,8-HexaCDF	26	21	19	6.2	86	26	22	19	5.2	74	0.534
2,3,4,6,7,8-HexaCDF	1.2	1.0	0.7	1.0	3.4	1.2	1.0	0.6	1.0	3.4	1.000
1,2,3,7,8,9-HexaCDF	ND					ND					
1,2,3,4,6,7,8-HeptaCDF	2.2	1.0	1.5	1.0	6.5	2.3	1.0	1.8	1.0	7.8	0.820
1,2,3,4,7,8,9-HeptaCDF	ND					ND					
OctaCDF	ND					ND					
Total PCDF	342	280	229	71	963	344	292	230	71	890	0.732
33'4'4'-TriCB(#77)	6.9	5.0	3.7	5.0	16	8.9	7.5	4.3	5.0	20	0.016
344'5-TriCB(#81)	5.3	5.0	1.4	5.0	12	5.7	5.0	2.4	5.0	15	0.180
33'44'5-PentaCB(#126)	129	100	81	30	391	131	96	85	34	356	0.770
33'44'55'-HexaCB(169)	279	250	144	104	678	293	280	129	114	585	0.101
Total Non-ortho PCBs	420	382	178	183	906	439	406	166	196	789	0.078
233'44'-PentaCB(#105)	4454	3145	3555	1206	13788	4581	3236	3714	5.0	15228	0.501
2344'5-PentaCB(#114)	2800	2365	1688	5.0	7194	2997	2681	1699	5.0	6987	0.118
23'44'5-PentaCB(#118)	21718	16568	17601	5.0	75475	21050	15412	14335	4575	57260	0.990
2'344'5-PentaCB(#123)	304	228	273	5.0	1239	312	214	237	5.0	898	0.581
233'44'5-HexaCB(#156)	50472	32661	46375	13079	195017	51038	30741	43667	9528	180163	0.517
233'44'5'-HexaCB(#157)	13157	8088	13150	3390	53954	12747	7644	11520	2332	46994	0.990
23'44'55'-HexaCB(#167)	4834	4243	3373	5.0	16863	4610	4265	2422	985	10481	0.770
233'44'55'-HeptaCB(#189)	7385	5100	5888	1664	24429	7398	5397	5323	1730	22434	0.829
Total Mono-ortho PCBs	105125	83472	66740	40066	293077	104734	93659	59308	34746	267273	0.829
TEQ from PCDDs	24	24	7.9	11	43	24	24	9.2	11	42	0.809
TEQ from PCDFs	82	63	54	16	223	82	68	54	16	211	0.534
TEQ from PCDDs/PCDFs	106	83	60	27	265	107	87	61	28	249	0.790
TEQ from non-*ortho* PCBs	21	20	9.8	7.4	54	22	20	9.7	7.8	47	0.485
TEQ from mono-*ortho* PCBs	3.2	2.5	2.0	1.2	8.8	3.1	2.8	1.8	1.0	8.0	0.829
TEQ from dioxin-like PCBs	24	22	11	9.0	60	25	23	11	9.1	51	0.603
Total TEQ	130	120	65	42	303	132	117	65	43	283	0.869

ND (less than the detection limit) values introduced to half values of the detection limit and calculated the TEQ concentrations

*SD* standard deviation, *CDD* chlorinated dibenzo-p-dioxin, *CDF* chlorinated dibenzofuran

**Table 2:** Effect of colestimide on the individual congener concentrations of non-dioxin-like PCBs in the blood of Yusho patients

IUPAC#	Concentration (pg/g lipid)										*p* Values
	Before the clinical trial					After the clinical trial					
	Mean	Median	SD	Minimum	Maximum	Mean	Median	SD	Minimum	Maximum	
TriCB-28	1644	1449	866	324	3809	1837	1866	1226	5	6187	0.025
TriCB-29	20	12	18	5	72	20	5	23	5	99	0.845
TriCB-37	128	5	245	5	847	73	5	165	5	698	0.112
TeteraCB-44	348	248	523	5	2841	415	324	415	107	2261	0.034
TeteraCB-47/48	525	359	437	117	1769	640	471	715	121	3659	0.049
TeteraCB-49	295	179	409	44	1679	344	216	576	5	3070	0.101
TeteraCB-52/69	956	780	836	294	4572	1060	860	745	368	3896	0.052
TeteraCB-56/60	442	306	344	5	1412	489	284	577	104	3010	0.889
TeteraCB-63	116	117	65	5	280	140	118	69	5	360	0.382
TeteraCB-66	2118	1520	1507	586	5853	2181	1536	1691	613	8475	0.551
TeteraCB-70	362	130	807	13	3375	418	143	1308	55	6817	0.280
TeteraCB-71	37	11	56	5	238	126	5	490	5	2524	0.586
TeteraCB-74	14823	12720	9202	3830	41089	14505	11875	9068	2973	35194	0.770
PentaCB-85	247	139	335	5	1592	205	138	218	5	1086	0.657
PentaCB-87	812	797	448	5	1716	747	697	442	5	2059	0.183
PentaCB-92	719	571	482	5	2402	752	669	455	5	2264	0.412
PentaCB-93/95/98	727	637	439	5	1964	1003	746	1165	326	6428	0.258
PentaCB-99	23623	19114	17453	4240	90685	24873	23328	16634	4308	82151	0.182
PentaCB-101	1931	1534	1234	5	5667	2337	1959	1481	600	6915	0.174
PentaCB-107/108	963	785	707	5	3340	961	819	584	5	2435	0.166
PentaCB-110	339	242	325	5	1451	332	298	268	5	1428	0.638
PentaCB-117	1911	1466	1813	435	7951	1722	1306	1642	5	6579	0.280
HexaCB-128	925	685	660	5	3099	949	678	775	5	3899	0.443
HexaCB-130	7065	5603	5780	2080	25122	7238	5886	5578	1913	25258	0.568
HexaCB-132	399	326	252	5	1125	445	397	282	5	1134	0.143
HexaCB-134	25	5	50	5	183	35	5	47	5	168	0.203
HexaCB-135	419	342	318	5	1577	485	330	403	5	1587	0.382
HexaCB-137	10565	7132	9066	2996	41244	10646	7786	8734	2336	39991	0.889
HexaCB-138	96984	89163	52967	25546	240863	97685	84306	53897	23381	244647	0.990
HexaCB-139/149	635	452	619	15	2404	615	292	696	5	2303	0.568
HexaCB-141	328	255	246	5	1044	340	282	287	5	1169	0.716
HexaCB-146	32968	34220	16346	11603	83149	35211	31688	16262	9839	68936	0.086
HexaCB-147	724	567	463	5	1678	768	622	519	5	1806	0.527
HexaCB-151	1329	981	880	428	3402	1349	1008	1098	5	4265	0.258
HexaCB-153	200929	184176	106109	73832	516088	206380	180663	109234	59314	458743	0.501
HexaCB-163/164	48797	47157	25168	17426	113577	49567	47872	22738	15767	88552	0.694
HexaCB-165	ND					ND					
HeptaCB-170	69704	60801	39523	16403	194289	69053	62645	37071	9946	142922	0.829
HeptaCB-172	9947	9273	5381	2768	27207	10156	10553	5277	5	20365	0.354
HeptaCB-177	15845	14513	9212	4504	39642	16359	14681	9932	2598	40496	0.424
HeptaCB-178	15885	13134	11788	5	56211	17209	13068	11123	3710	42945	0.012
HeptaCB-179	281	182	296	5	1110	357	202	384	5	1463	0.005
HeptaCB-180	205779	201272	136971	50473	703408	203297	188508	121684	32744	490934	0.970
HeptaCB-181	553	292	671	5	2776	581	296	716	5	2806	0.264
HeptaCB-182/187	76063	60684	61028	14834	270253	81845	61046	63293	14093	238587	0.019
HeptaCB-183	16843	14980	12005	4733	45012	17864	15557	13292	3756	55788	0.182
HeptaCB-191	3078	2922	2008	5	8667	2907	2762	1688	805	7561	0.280
OctaCB-194	31774	32293	22776	5	116675	32519	32465	19427	8258	84219	0.304
OctaCB-195	7832	6835	5594	5	26180	7929	7156	4776	1879	19564	0.381
OctaCB-196/203	17107	15138	11346	5	55263	16821	15312	9331	4346	38617	0.869
OctaCB-198/201	14771	12536	11520	5	56995	14368	11829	8673	4251	34263	0.534
OctaCB-200	659	485	607	5	2350	705	593	609	5	2449	0.083
OctaCB-202	5432	3893	4532	5	22569	5298	3892	3307	1509	12550	0.258
OctaCB-205	977	898	633	5	2795	969	912	456	289	1949	0.770
NonaCB-206	5049	4561	2829	1502	14874	4891	4639	2446	5	10726	0.657
NonaCB-207	922	755	572	5	2501	911	792	522	5	1905	0.326
NonaCB-208	1877	1731	1209	5	6338	1827	1723	953	5	4559	0.778
DecaCB-209	1857	1598	890	893	5115	1900	1946	730	837	4005	0.200
Total TrCBs	1792	1471	999	334	3921	1931	1917	1198	708	6225	0.182
Total TeCBs	20023	17013	11246	6548	50619	20318	16713	11351	5981	43194	0.424
Total PeCBs	31271	26027	19945	7137	102693	32932	31001	19076	7680	93210	0.228
Total HxCBs	402098	373141	196792	152976	892316	411718	358344	198664	131539	769040	0.675
Total HpCBs	413979	401285	265364	105126	1341206	419627	378119	250647	80774	971840	0.620
Total OcCBs	78553	77549	55605	35	281931	78608	77101	44919	21477	186948	0.409
Total NoCBs	7849	6439	4416	2355	23712	7629	7224	3832	15	17159	0.869
Total DeCBs	1857	1598	890	893	5115	1900	1946	730	837	4005	0.200
Total PCBs	957422	871523	520304	320807	2642555	974664	806289	495089	286088	2006817	0.585

ND (less than the detection limit) values introduced to half values of the detection limit and calculated the TEQ concentrations

*SD* standard deviation, *CB* chlorinated biphenyl

The arithmetic mean TEQ concentrations of PCDDs, PCDFs, non-*ortho* PCBs, and mono-*ortho* PCBs in the blood of the 26 Yusho patients were 24, 82, 21, and 3.2 pg TEQ/g lipid, respectively, before the trial, and 24, 82, 22, and 3.1 pg TEQ/g lipid, respectively, after the trial. Total TEQ concentration of these dioxin-like compounds equaled 42–303 (mean: 130, median: 120) pg TEQ/g lipid before the trial, and 43–283 (mean: 132, median: 118) pg TEQ/g lipid after the trial, indicating that the concentrations before the trial were almost the same as those after the trial. Regarding the non-dioxin-like PCB concentrations, the sums of the concentrations of 58 PCB congeners in the blood before and after the trial were 321–2643 (mean: 957, median: 872) and 286–2007 (mean: 975, median: 806) ng/g lipid, respectively. The arithmetic mean concentrations of triCBs, tetraCBs, pentaCBs, hexaCBs, heptaCBs, octaCBs, and nonaCBs in the blood of Yusho patients were 1.8, 20, 31, 402, 414, 79, and 7.8 ng/g lipid, respectively, before the trial, and 1.9, 20, 33, 412, 420, 79, and 7.6 ng/g lipid, respectively, after the trial, indicating that concentrations of these PCBs compounds were also almost the same before and after the trial. These results indicated that the concentrations of PCDDs, PCDFs, dioxin-like PCBs and non-dioxin-like PCBs in the blood of Yusho patients were not significantly altered by the intervention with oral colestimide.

We previously reported that the concentrations of 1,2,3,6,7,8-hexaCDD, 2,3,4,7,8-pentaCDF, 1,2,3,4,7,8-hexaCDF, 1,2,3,6,7,8-hexaCDF, hexaCB-169, hexaCB-156, hexaCB-157, and heptaCB-189 in the blood of Yusho patients were higher than those of the normal controls [8, 9]. These can be considered the characteristic congeners in the blood of Yusho patients. 2,3,4,7,8-PentaCDF is recognized as the most important causative agent for subjective symptoms of Yusho. Blood levels before and after the trial were 48–636 (mean: 241, median: 191) and 49–613 (mean: 242, median: 205) pg TEQ/g lipid, respectively, indicating that the concentration did not significantly decrease with administration of colestimide. This was also the case for the concentrations of other characteristic congeners before and after the trial. Among congeners of PCDDs, PCDFs, dioxin-like PCBs, and non-dioxin-like PCBs, most congeners did not show statistically significant differences. According to these results, the therapeutic usefulness of colestimide in reducing the concentrations of PCDDs, PCDFs, and PCBs in blood of Yusho patients could not be confirmed.

## Discussion

Over 48 years have passed since the outbreak of Yusho disease. However, some patients are still afflicted with intractable symptoms such as chloracne, general fatigue and neuropathy [12]. There are patients who continue to have much higher concentrations of dioxin-like compounds in their blood than unaffected persons. Moreover, the half-lives of blood concentrations of 2,3,4,7,8-pentaCDF have become long to near infinity in the majority of Yusho patients [24]. To reduce the concentrations of PCDDs, PCDFs, and PCBs in the blood of Yusho patients, our study group previously conducted a clinical trial using cholestyramine and rice bran fiber [19, 20]. Results of that study showed that the amounts of 2,3,4,7,8-pentaCDF in patients’ feces actually increased, although beneficial clinical effects were not apparent, possibly due to a short trial period. A recent study reported that colestimide can decrease the concentrations of PCDDs, PCDFs, and PCBs in blood [21, 22]. Eight male and two female healthy subjects were treated with colestimide (3 g/day) for 6 months. In this report, colestimide was effective for promoting excretion of dioxin-like compounds from the human body. Colestimide is a non-absorbable anion exchange resin and enhances excretion of cholesterol in feces by inhibiting absorption of food-derived cholesterol in the intestinal tract [25]. Based on this result, we designed a clinical trial with colestimide for Yusho patients. However, in the present study, we were unable to confirm a significant decrease in most congeners of PCDDs, PCDFs, and PCBs in the blood of Yusho patients. It is suggested that the PCDDs, PCDFs, and PCBs that have remained in the whole body of patients over the 45 years since the outbreak of Yusho are very difficult to excrete from the body. In the present trial, there may be many limitations such as a small number of participants, duration of administration period and dose of cholestimide. Out of the 36 patients who participated in the trial, 9 patients experienced serious adverse effects (constipation or abdominal distension) by the repeated administration of colestimide. Therefore, we cannot recommend that elderly patients participate in clinical trial studies for such long periods as in the present study.

## Conclusion

Although over 48 years have passed since the outbreak of Yusho, many patients still suffer various symptoms such as chloracne, general fatigue and neuropathy. The concentrations of causative dioxin-like compounds in their blood remain at high levels. We examined whether oral administration of colestimide could reduce the concentrations of PCDDs, PCDFs, and PCBs in the blood of Yusho patients. However, the effectiveness of colestimide on the concentrations of these dioxin-like compounds in the blood of Yusho patients could not be confirmed.

## Abbreviations

PCDDs, polychlorinated dibenzo-*p*-dioxins; PCDFs, polychlorinated dibenzofurans; PCBs, polychlorinated biphenyls; WHO, World health oganization; TEQ, toxic equivalent; TEF, toxic equivalency factor
